# The effect of long-term systemic immunosuppressive drug use on druse formation: a new perspective to age-related macular degeneration

**DOI:** 10.3906/sag-2001-36

**Published:** 2020-12-17

**Authors:** Özkan SEVER, Rıdvan MERCAN

**Affiliations:** 1 Department of Ophthalmology, Faculty of Medicine, Namık Kemal University, Tekirdağ Turkey; 2 Department of Rheumatology, Faculty of Medicine, Namık Kemal University, Tekirdağ Turkey

**Keywords:** Druse, systemic immunosuppression, inflammation, age-related macular degeneration

## Abstract

**Background/aim:**

To evaluate the effect of the long-term use of systemic immunosuppressive drugs on druse formation in patients aged over 50 years.

**Materials and methods:**

The current retrospective cohort study includes 420 eyes of 420 patients. 210 eyes of 210 patients who used immunosuppressive drugs (Group 1) at least for the last 5 years and 210 eyes of 210 control patients (Group 2) who did not use any drugs were compared. All patients were older than 50 years and selected among patients who were followed by rheumatology and ophthalmology clinic at a tertiary university hospital. All patients had complete ophthalmic examination, fundus photography and optical coherence tomography (OCT). The primary outcome of this study is the difference in macular and paramacular druse formation rates between two groups.

**Results:**

Small, intermediate, large, soft, and paramacular druse formation rates were significantly lower in Group 1 than those in Group 2 (P = 0.028, P = 0.001, P = 0.001, P = 0.001, and P = 0.001, respectively).

**Conclusion:**

Patients who used long-term systemic immunosuppressive drugs had significantly lower hard and soft druse formation rate than age and sex matched control subjects.

## 1. Introduction

Globally, age-related macular degeneration (AMD) accounts for 8.7% of all blindness cases and is the leading cause of irreversible visual impairment among individuals aged ≥65 living in developed countries [1,2]. AMD is characterized by extracellular material, collectively described as druse, which mostly accumulates under the retina pigment epithelium (RPE). The mechanism underlying the origin and growth of druse remains unclear. Researches focused on lipids and minerals for druse formation; however, there is relatively less information regarding the origin of drusen associated proteins and how they are retained in the space between the basal lamina of the RPE and the inner collagenous layer. Considered to be an early stage of AMD, druse formation involves the accumulation of intracellular lipofuscin in the RPE and the build-up of extracellular deposits under the RPE. It is widely accepted that this local accumulation upregulates cytokines and acute phase reactants, which activate the complement cascade, and RPE and local cells respond to the attack by complement proteins. Finally the dendritic cells in the choroid invade incipient druse, and a strong immune response is initiated against exposed antigens in the sub-RPE [3]. While some authors suggest that druse proteins primarily originate from the cellular debris of the outer segment of processed photoreceptors and RPE, others suggest a choroidal cell or blood-derived origin [4]. The commonly accepted pathological pathway involves the oxidative modification of these lipids, resulting in cross-linking of these molecules that leads to deposit and subsequently druse formation. Local cellular damage at the very early onset of AMD, via complement cascade attack on druse proteins may lead to retinal damage and more advanced AMD [5].

Many studies have reported on the outcomes of treated or untreated AMD, including geographic atrophy or choroidal neovascularization. Although drusen, regardless of its subtypes, are widely accepted as the early finding of AMD, the prevention and treatment of these lesions remain unknown. Studies reported a correlation between complement system activation and AMD.  This is based on histological and proteomic data, showing complement proteins in druse of postmortem eyes [6–8].

In the current study, we aimed to evaluate the effect of long-term use of systemic immunosuppressive drugs on druse formation and subsequently, the impact of antiinflammation on the formation of these early lesions.

## 2. Methods

This study was conducted in accordance with the tenets of the Declaration of Helsinki and was approved by the Local Ethical Committee of the same research hospital. This is a retrospective cohort study, and the participants were selected among the patients visiting the rheumatology and ophthalmology clinic of a tertiary referral clinic from Turkey. Systemic immunosuppressive drug users were chosen among patients who were sent to ophthalmology clinic for fundus examination before initiation of the treatment to rule out any contraindication for use of systemic immunosuppressive drugs and to confirm the absence of any drusen. All patients were Caucasian. Control group patients were chosen among the patients who had visited ophthalmology clinic and in the last 10 years who had no druse. Their mean follow-up duration was (7.1 ± 1.3 years). All study and control patients had fundus photographs within ten years before the study. Patients who used immunosuppressive drugs for over 5 years were included in Group 1; age, sex-matched control patients with no history of immunosuppressive drug usage or AMD were included in Group 2. Smoking more than 10 cigarettes a day, glaucoma, diabetic retinopathy and any corneal lesions were among exclusion criteria. All patients in the group 1 had rheumatoid arthritis or ankylosing spondylitis for the last ≥5 (5–18) years. Complement system activation was accepted as a risk factor of druse formation. Therefore, we included only those patients who used immunosuppressive drugs that inhibit the complement system and cytokine activation including azathioprine, cyclosporine, leflunomide, methotrexate, and oral steroids.

We classified drusen as small (diameter, <63 µm), intermediate (diameter, 63–125 µm), large (diameter, >125 µm), and soft (diameter, >125 µm with frequently more ill-defined edges) [9].

**Figure 1:**
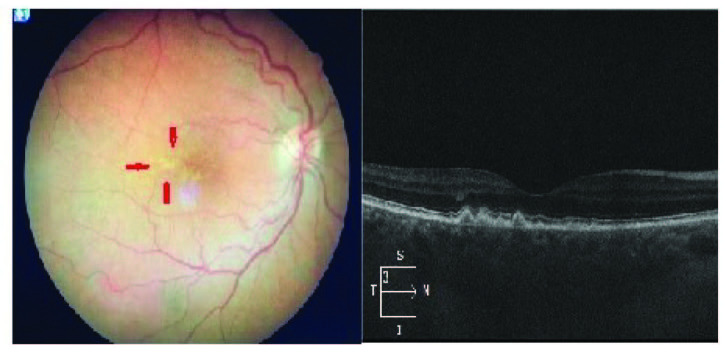
Colour fundus photo and optical coherence tomography of a large macular druse from Group 1.

Fundus photographs (Zeiss F-450) and optical coherence tomography (Cirrus HD-OCT, Carl Zeiss Ophthalmic System Inc., Zeiss-Humphrey, Dublin, CA, USA) scans were obtained by two different retina specialist. The only information available to the operators was the age of the patients. The images were randomized to allow each eye to be independently graded in accordance with inclusion of a single eye from each patient/control. Druse sizes were measured manually by the graders. Paramacular area was decided as retinal areas outside the clinical macula which is accepted as 1.5 mm diameter around fovea. Figure 1 shows a color fundus photo and an OCT image of a large druse from Group 1. For quality control, all images were graded by two graders; any difference was resolved by a third, senior grader.

The primary outcomes included macular and paramacular druse formation. Secondary outcome measures included visual acuity, intraocular pressure (IOP), cataract formation, and the mean time of immunosuppressive drug use.

### 2.1. Statistical analysis

Data were expressed as means ± standard deviations and medians. Data distribution was assessed using the Kolmogorov–Smirnov test. For comparison of the continuous and independent variables Mann–Whitney U test was used. For comparison of categorical variables (especially evaluation of druse and cataract formation, hypertension, family history, cigarette smoking) the chi-square test was used. A p value of <0.05 was accepted as statistically significant. Statistical analysis was performed using a licensed statistical software (IBM SPSS version 22.0; IBM Corp., Armonk, NY, USA).

## 3. Results

This cross-sectional study included 420 eyes of 420 patients: Group 1 comprised 210 eyes of 210 patients, and group 2 comprised 210 eyes of 210 patients. The age of the patients was over 50 years in both the groups, and the median age was 61 (61.8 ± 5.8) years.

**Figure 2:**
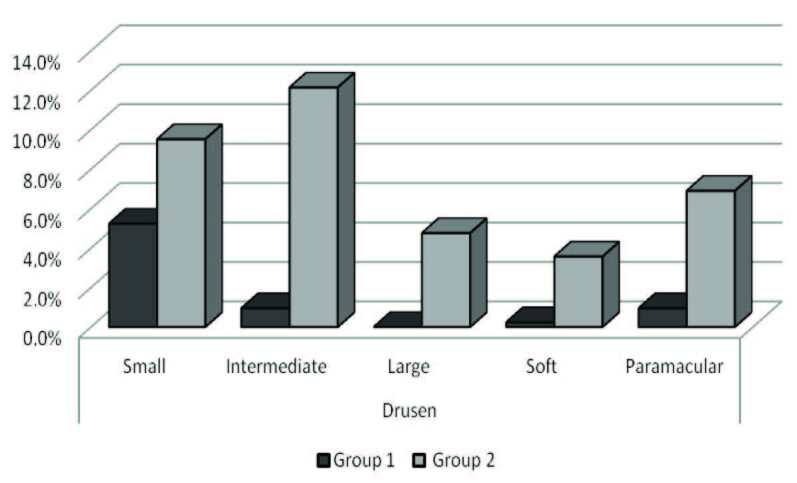
The druse formation rates of the groups. Group 1: the patients who used systemic immunosuppressive drugs, Group 2: control group who did not use any systemic immunosuppressive drugs.

The small, intermediate, large, soft, and paramacular druse formation rates were significantly lower in Group 1 than those in Group 2 (P = 0.028, P = 0.001, P = 0.001, P= 0.001, and P = 0.001, respectively). Druse formation rates in both groups were given in Figure 2. The table shows druse formation status and systemic effects between the two groups. Mean follow up time of the groups was 7.2 ± 1.9 (5–10) years. The mean time of immunosuppressive drug use among the patients in Group 1 was 7.1 ± 1.9 years. In Group 1, the mean age of the patients who developed any type of drusen was 56.7 ± 4.6 (57–63) years, and mean drug use time was 5.2 ± 1.2 (5–8) years, which is significantly shorter than those who used immunosuppressive drugs and developed no druse 7.3 ± 1.9 (5–10) years (P = 0.01). In Group 2, there was no patients who used immunosuppressive drugs and the mean age of patients who developed any type of drusen was 57.2 ± 3.2 (52–64) years.

**Table:** A comparison of the groups for druse formation, basic characteristics and external effects.

	Group 1	Group 2	P
Patient, n	210	210	1 X²
Age, mean ± sd	61.9 ± 6.0	60.8 ± 5.4	0.052 m
Sex, male, %	88 41.9%	88 41.9%	1.000 X²
IOP, mmHg, mean ± sd	14.4 ± 1.6	13.9 ± 1.3	0.001m
Cigarette smoking (<10 pieces\day), %	50 23.8%	57 27.1%	0.543 X²
Family history, %	35 16.6%	40 19%	0.352 X²
Hypertension, %	48 22.8%	57 27.1%	0.412 X²
Small drusen, %	12 5.7%	25 11.9%	0.028 X²
Intermediate drusen,%	4 1.9%	41 19.5%	0.001 X²
Large drusen, %	0 0.0%	16 7.6%	0.001 X²
Soft drusen, %	1 0.47%	10 4.7%	0.001 X²
Paramacular drusen,%	2 0.95%	19 9.0%	0.001 X²
Cataract, %	30 14.2%	4 1.9%	0.001 X²

m: Mann–Whitney U test; X²: chi-square test; IOP: intraocular pressure; Group 1: the patients who used immunosuppressive drugs; Group 2: control group who did not use any immunosuppressive drugs.

There was no statistically significant difference between the groups regarding age and sex (P = 0.052 and P = 1.0, respectively). Smoking rates of the groups were similar. Cigarette smoking rate in Group 1 was 23.8% (50\210), while 27.1 % (57\210) of Group 2 was smoking at most 10 pieces of cigarette (P = 0.543). IOP of group 1 (14.4 ± 1.6 mmHg) was significantly higher than that of group 2 (13.9 ± 1.3 mmHg; P = 0.001). However, none of the patients had glaucoma. Overall, 42 eyes (10%) in Group 1 and 30 (7.1%) in Group 2 were pseudophakic (P = 0.23); 30 eyes (14.2%) in Group 1 and 4 (1.9 %) in Group 2 had cataract; and this difference was statistically significant (P = 0.001). Eleven patients (2.9%) from Group 1 had a history of uveitis. This low rate of uveitis might be a result of strong immunosuppression since the beginning of diagnosis of the diseases. In Group 1, 85 (40.1%), 150 (71.4%), 95 (45.2%), 92 (43.8%), and 71 patients (33.8%) were using steroids, methotrexate, leflunomide, azathioprine, and cyclosporine, respectively. Furthermore, 109 (66.1%) and 81 (38.5%) patients were using three and two different immunosuppressive drugs, respectively. In each group, more than one drug was used in the last ≥5 (7.1 ± 1.9) years. Mean duration of drug usage was 5.7, 8.3, 4.1, 7.3, and 5.8 years for steroids, methotrexate, leflunomide, azathioprine, and cyclosporine, respectively.

## 4. Discussion

In this study, we show that systemic immunosuppression had been associated in our patient cohort with both slower hard and soft druse formation rates. There are only a few studies which evaluate association of systemic immunosuppressive drugs and druse formation. Furthermore, in this study we focused on the efficacy of any therapeutic agent in druse formation. Drusen are accepted as the hallmark of AMD. However, several questions remain unanswered, including the source of origin of the druse protein; it is unclear whether these proteins originate from the photoreceptors, RPE, choroidal endothelium or circulating blood. Moreover, it is unknown whether druse formation can be prevented. The most accepted mechanism of druse formation involves the thickening of the Bruch’s membrane with advancing age, the membrane becomes increasingly impermeable to low concentrations of waste products that begin to accumulate in the sub-RPE space. Although age is an independent risk factor of druse formation, inflammation has been reported to accelerate this process [10]. Our results strongly suggest that druse formation may result from local and/or systemic inflammation and is not merely due to lipid deposition on the thickened Bruch’s membrane.

There are two prominent genetic locations, complement factor H (CFH) and age-related maculopathy susceptibility 2 (ARMS2), which creates susceptibility to AMD. The CFH gene encodes for complement factor H, a glycoprotein that plays an integral role in the regulation of the alternative complement pathway and thought to be responsible from dry type AMD. The ARMS2 gene triggers the complement system at the surface of retinal monocytes and microglia by binding to the surface of the apoptotic and necrotic cells and primarily responsible from wet type AMD [10–13]. Both types of AMD are strongly related with complement system activation. A previous study reported that compared with low-risk eyes (homozygous for Y402), high-risk eyes (homozygous for the Y402H SNP) demonstrated greater C-reactive protein (CRP) immunoreactivity in the choroid, particularly in regions containing druse-like deposits [14]. Seddon et al. demonstrated that CRP levels and CFH genotype were independently associated with AMD risk [15]. A strong inflammatory subject is smoking and increases the risk of AMD for all CFH, ARMS2, and HtrA serine peptidase 1 (HTRA1) genotypes [16,17]. These pathways show the relevance of inflammation on AMD and compel us to find a way to prevent inflammation. A recent study proposed therapeutically targeting systemic properdin and therefore ameliorating the alternative complement system with could be effective to treat locally complement-mediated diseases [18]. On the other hand, to the best of our knowledge, no study till date has reported the effect of inflammation on the pathway prior to druse formation as there exists no predictive biomarker before druse formation.

Uveitis is a common manifestation of systemic inflammatory diseases and it has been reported that patients with uveitis have lower rate of druse formation. Investigators report that this sparing from AMD in uveitis might be a result of long-term immunomodulatory treatment [19]. Besides in our study we report that the duration of drug use was strongly correlated with lower druse rates. The patients in Group 1, who used systemic immunosuppressive drugs for a longer period had lower druse formation rates. Recently some studies investigated early-onset macular degeneration (EOMD) to determine a predictive marker of early AMD and druse formation. A study investigating EOMD in seven families reported that the FHL-1 gene, which is suggested to be the main regulator of complement cascade in the Bruch’s membrane, causes complement turnover dysregulation in the membrane, complement overactivation, and EOMD development [20]. The age-related eye disease study 1 (AREDS1) reported that the antioxidants, vitamins C, vitamin E, beta carotene, and zinc reduce the risk of progression to advanced AMD. The age-related eye disease study 2 (AREDS2), which included 4203 patients from 82 clinics, reported that the addition of lutein + zeaxanthin, omega-3 long-chain polyunsaturated fatty acids [docosahexaenoic acid (DHA) + eicosapentaenoic acid (EPA)] or lutein + zeaxanthin and DHA + EPA to the AREDS1 formulation in the primary analyses did not further reduce the risk of progression to advanced AMD [21]. AREDS2 reported that lutein + zeaxanthin require further investigation for their potential inclusion in the AREDS supplements. The limitations of AREDS2 include inadequate dose, inadequate duration of treatment, or both. Currently, antioxidation seems to be the hallmark for preventing the progression of dry AMD [16].

Systemic immunosuppressive drugs have different effects on immune system and AMD. Corticosteroids inhibit proinflammatory cytokines, decrease choroidal permeability and lower vascular endothelium growth factor (VEGF) levels [22]. Cyclosporine directly inhibits T cells which play a crucial role in AMD pathogenesis [23]. Leflunomide acts as an inhibitor of aryl hydrocarbon receptor which is a ligand activated transcription factor and plays a role in AMD pathogenesis [24]. Methotrexate basically inhibits TNF-α synthesis and T lymphocyte proliferation by means of reducing new purine and pyrimidine synthesis [25,26]. Also there are many studies about the effect of hypercholesterolemia and statin use in AMD pathogenesis. Some of them reported that hypercholesterolemia increased the rate of AMD and some reported that statins lower the rate of AMD diagnosis however there is still no certain proof in the literature [27,28].

Between 2020 and 2040, AMD is estimated to affect approximately 200 million people globally and approximately 2.8 million in Europe; two-thirds of this population will have neovascular AMD, requiring multiple anti-VEGF treatments [2,29]. In accordance with the previous studies, the optional number of intravitreal anti-VEGF injection ranges between 8 and 11 in a year. In contrast, in the real-world setting, the number of injections is far lower with a maximum six injections per year [30,31]. A study assessing the cost-effectiveness of such treatments in the United Kingdom reported the annual cost of ranibizumab and aflibercept for AMD as €33,000 and € 31,000, respectively, and this cost is continuously increasing [32]. On the other hand annual cost of a patient with rheumatoid arthritis with anti-TNFs and/or methotrexate/corticosteroids will be €39,000 for all systemic problems [33]. Currently, especially for developing countries, we either have to find new, cost-effective treatment solutions or have to identify a way to prevent the disease.

Studies reported that rheumatoid arthritis and HLA-B27 positive diseases increase the AMD diagnosis [34, 35]. In our study we report that the effect of immunosuppression is not only confined with decreasing the increased risk of AMD in these inflammatory diseases but also decreases the risk of druse formation even compared with normal population.

A study assessing the origin of druse proteins reported that these proteins primarily originate from the blood and less commonly from the RPE, photoreceptors, or choroid [36]. We propose that if we can prevent systemic drusenomics, we can lower druse formation. Our results suggest that druse may not be limited to local lipofuscin accumulation and is rather a result of oxidative stress, which may be prevented using immunosuppressive drugs. Patient characteristics, including family history, high serum CRP levels and old age may be considered as the potential risk factors of druse formation. These risk factors could be considered to investigate the genetic tendency of these patients toward the development of AMD; moreover, it may be reasonable for patients with ophthalmological signs and proved genetic tendency of AMD to undergo low dose systemic immunosuppressive drug therapy. We believe this will not only decrease costs but also decrease morbidity.

Cataract formation of Group 1 was significantly higher than the other group. Although 40 % of the patients in Group 1 were on systemic steroids only 14.2% of them developed cataract. IOP of the patients in Group 1 was higher than Group 2 however no one developed glaucoma.

The major limitations of our study are absence of fundus autoflourescence photos of the patients, and retrospective study design.

In conclusion, immunoreactivity appears to be the reason for druse and AMD formation and certain immunosuppressive or immunomodulatory drugs at a reasonable dose may prevent druse formation or progression of AMD. Ocular side effects of the patients were evaluated as reasonable. However, patients should be careful about systemic side effects while using systemic immunosuppressive drugs. This study needs to be further supported by prospective and large-scale studies.

## Informed consent

This study received institutional review board approval from Namık Kemal University Local Ethics Committee and all provided informed consent in the format required by the relevant authorities. Ethical approval number is 2019.18.02.02.
